# Efficacy of Thiamine and Medical Management in Treating Hyperuricemia in AUD Patients with ALD: Role of Hyperuricemia in Liver Injury, Gut-Barrier Dysfunction, and Inflammation

**Published:** 2021-07-28

**Authors:** Vatsalya Vatsalya, Fengyuan Li, Jane Frimodig, Nihar Shah, Amar Sutrawe, Wenke Feng

**Affiliations:** 1 Department of Medicine, University of Louisville, Louisville, Kentucky; 2 Alcohol Research Center, University of Louisville, Louisville, Kentucky; 3 Robley Rex Louisville VA Medical Center, Louisville, Kentucky; 4 Department of Pharmacology and Toxicology, University of Louisville, Louisville, KentuckyUSA

**Keywords:** Hepatocytic cell, Cytokeratin, Pro inflammatory, Steatohepatitis, Hyperuricemia

## Abstract

**Background:**

Hyperuricemia has been reported in liver injury; however its role in the early stage of Alcohol-associated Liver Disease (ALD) has not been examined yet. This study investigated the role of Serum Uric Acid (SUA) in alcohol-related liver disease, gut barrier dysfunction, and inflammation activity. This study also evaluated the efficacy of abstinence, treatment with thiamine and medical management to alleviate hyperuricemia.

**Methods:**

48 heavy drinking Alcohol Use Disorder (AUD) patients (34 males [M]/14 females [F]) participated in this study. Patients were grouped by serum Alanine Aminotransferase (ALT) levels as group 1 (ALT ≤ 40 U/L, 7M/8F) and group 2 (ALT>41U/L, 27M/6F). All patients received open label thiamine 200 mg daily dose. Demographics, drinking history (using Lifetime Drinking History [LTDH], and Timeline Follow Back [TLFB] for the past 90 days) reports were collected at baseline. Baseline and three-week assessments for SUA, biomarkers of liver injury, endotoxemia and inflammation were evaluated.

**Results:**

22 out of 48 AUD patients reported hyperuricemia, primarily in males. SUA was significantly associated with ALT in each group (in group 2, when covaried with HDD90). SUA was also significantly associated with gut barrier dysfunction markers, LBP and LPS, in group 2, SUA and LBP predicted IL-1**β** significantly in group 2. Uric acid along with IL-1**β** and HDD90 significantly predicted necrotic type of hepatocyte cell death in group 2. Post-treatment SUA dropped across both the groups, significantly in females; adverse effects of drinking, cytokine and uric acid interaction on liver cell death also decreased in group 2. *In vitro* experiments validated the efficacy of thiamine on hepatocytic uric acid production in alcohol sensitization.

**Conclusion:**

Uric acid, a metabolic risk signal, was likely involved in the interaction of proinflammatory activity with heavy drinking markers at early-stage ALD. Three-week inpatient medical management, along with treatment with thiamine, seems to alleviate baseline hyperuricemia and necrotic type of hepatocytic cell death in AUD patients with liver injury.

## INTRODUCTION

Excessive alcohol intake could lead to a spectrum of disease conditions diagnosed broadly as Alcohol-associated Liver Disease (ALD), ranging from steatosis, steatohepatitis, foamy degeneration, fatty liver with cholestasis, to hepatitis and cirrhosis [1,2]. The advanced or progressed form of ALD has been widely characterized; however, little is known about the early stage of ALD [3]. Lack of specific biomarkers that could characterize early ALD remains a gap in the understanding of pathology and progression of the disease and has potential as a therapeutic target.

Recent findings suggest that elevated SUA is associated with a variety of other systemic conditions, for example: hypertension, Kidney disease, metabolic syndrome, cardiovascular disease and type 2 diabetes. SUA has been studied with respect to alcohol intake and metabolism for some time [4-6]. One study suggested that higher Serum Uric Acid (SUA) is indicative of alcohol abuse [7]. However, the role of UA in early stage ALD in humans has not been studied, neither in terms of its association with heavy drinking markers, or with liver injury measures. Higher UA or hyperuricemia has been long associated with gout disease [8]. In liver studies, elevated SUA has shown a close association with non-alcoholic fatty liver disease independent of the identification of metabolic syndrome [9]. Nonetheless, SUA changes and its treatment in ALD (in humans) remains an understudied area of investigation.

Some clinical and preclinical studies have shown efficacy of thiamine in reducing uric acid levels in diabetic rats, and hyperaminoacidemia in humans [10,11]. A potential catabolic pathway of adenosine monophosphate involves in the synthesis of majority of the UA that is the final product of purine oxidative metabolism and is excreted through urine. Thiamine diphosphate could scavenge adenosine monophosphate, which is found in liver tissue [12-14]. Notably, Benfotiamine which is a derivative of thiamine at 4 week low dose (70 mg/kg/day) regimen reduces the effects of uric acid by improving the serum concentration of nitrite/nitrates [2]. Thus, thiamine could reduce a substrate of the upregulated catabolic pathway involved in UA synthesis. However, the role of thiamine in lowering UA levels longitudinally in AUD patients has not previously been evaluated.

Higher SUA is related to higher levels of circulating inflammatory cytokines in systemic inflammation, though their interactions have not been tested in early ALD. IL-22 generally promotes liver repair whereas IL-17 mediates liver injury, and the expression profiles of these mutually antagonistic cytokines shift in favor of IL-17 in advanced stage [15]. Anti-oxidant properties of thiamine have been reported previously, and it is administered in alcohol dependent patients to treat confusion, vision impairment, and memory loss that results from acute thiamine deficiency [1,16]. Thus, testing the role of thiamine in reducing proinflammatory activity in early ALD is important.

In this study, we aim to characterize SUA with the markers of heavy drinking, cytokine response, and liver injury in Alcohol Use Disorder (AUD) patients who exhibited either no liver injury or mild liver injury. We also evaluated the differences in response to 3-week detox and thiamine treatment for alleviation of SUA, and corresponding liver injury and pro-inflammatory activity. To experimentally verify the clinical findings, we tested the effects of thiamine (described as VB1 in the experimental design).

## MATERIALS AND METHODS

This investigation is a secondary aim of a larger clinical investigation (NCT#00106106) that was conducted at the National Institute on Alcohol Abuse and Alcoholism (NIAAA) at the National Institutes of Health (NIH), Bethesda MD. Study was approved by the central neuroscience IRB committee of the NIAAA. 48 male and female AUD patients between 21-65 years of age participated in this study. All study patients were diagnosed with AUD based on DSM-IV TR edition [16]. The alcohol dependence module of the structured clinical interview I and alcohol withdrawal were administered to diagnose AUD. Important exclusion criteria are described here: presence of severe psychiatric and/or somatic illnesses, including advanced lung disease, unstable cardiovascular disease (decompensation, as demonstrated through chest X-ray, pathological electrocardiogram), and/or renal failure (creatinine clearance <30 ml/min). Other exclusion criteria were: presence of HIV; pregnancy or ongoing breastfeeding; and pronounced anxiety provoked by enclosed spaces, and/or positive urine screen for any illicit drug. No AUD patient exhibited any clinical evidence of advanced ALD, or gout disease. Further detailed information on admission, exclusion and inclusion and detox treatment can be reviewed in a primary publication on an investigational drug efficacy [17-19].

All study patients received daily doses of open label thiamine (100 mg twice daily) after the completion of the consenting process. All patients received standard clinical inpatient care for alcohol detoxification and medical management, including counseling according to the “Human Subjects Protection” guideline of NIH [20].

### Demographics, drinking and laboratory evaluations

Blood was drawn once patients consented to participate in this inpatient study. On admission, blood samples were collected for a serum chemistry panel that included tests for liver injury and the SUA level. Demographics (Age, Sex, Body Mass Index [BMI]) and drinking history information were also collected for the study. Heavy drinking measures were collected from the Time-line Follow-back questionnaire [21]. Markers of heavy drinking derived from TLFB reported in the past 90 days were “Total Drinks” (TD90), “Number of Drinking Days” (NDD90), “Number of Non-Drinking Days” (NNDD90), “Average Drinking per Drinking Days” (AvgDPD90), and “Heavy Drinking Days” (HDD90). We used “Controlling Nutritional Status Test” (CONUT) information on these patients to assess their nutritional status [22]. The alanine Aminotransaminase (ALT) level was used as a biomarker for early liver injury (Medline Plus-National Institutes of Health, 2014). Normal serum ALT values were set at<40 IU/L (based on the sample collection timing that corresponded with Medline Plus-NIH updates till 2014) and patients were categorized as group 1: those with normal ALT levels; and group 2: those with ALT>40 IU/L, as indicative of mild liver injury. The reference normal range for SUA was 2.6-6.0 mg/dL [23]. Patients with SUA>6.0 mg/dL were considered as having elevated levels (hyperuricemia) in relation to heavy alcohol drinking/AUD. All clinical laboratory tests were repeated by the end of 3^rd^ week (most of them on day 22). All clinical laboratory assays were performed by the Department of Laboratory Medicine at NIH Bethesda MD per its guideline.

### Laboratory assays

Frozen plasma samples at −80°Celsius were thawed and assayed. Plasma cytokeratin 18 whole protein (K18 M65) and caspase cleaved fragment (K18 M30) were analyzed using Enzyme-Linked Immunosorbent Assay (ELISA) (Peviva VLVbio, Nacka, Sweden) according to the manufacturer’s instructions. Clinically significant K18 is as following: K18 M65>500 U/l or CK18 M30>250 U/l. Plasma pro-inflammatory cytokines, TNF-**α**, interleukin 1**β**, interleukin 6, and interleukin 8 (1L1**β**, IL-6, and IL-8), PAI-1, and Monocyte Chemoattractant Protein-1 (MCP-1) were obtained by multianalyte chemiluminescent detection using Mulliplex kits (Millipore, Billerica, MA) on the Luminex (Luminex, Austin, TX) platform according to manufacturers’ instructions. Plasma Lipopolysaccharide (LPS) and LPS Binding Protein (LBP) levels were assayed using the kinetic chromogenic limulus amebocyte lysate assay (Lonza, Walkersville, MD) according to the manufacturer’s instructions.

### Analysis of IL-17 and IL-22 in a sub-set of AUD patients for designing proof-of-concept

We performed analyses for IL-17 and IL-22 on a sub-set of age and sex matched AUD patients (n=16) from this study’s original cohort (N=48), with the goal of developing an *in vitro* basic science experimental model to test the efficacy of thiamine. We also used n=8 as healthy volunteers in this study for comparison of IL-17 and IL-22 expression. Il-17 and Il-22 were detected in plasma using human Il-17A high sensitivity ELISA kits (BMS2017HS, Invitrogen) and human Il-22 ELISA Kits (BMS2047, Invitrogen) per the manufacturer’s instructions. Results were read on a Spectra Max plus 384 plate reader and modeled using their Soft Max Pro software (molecular devices, san jose, CA).

### Cell culture

Primary murine hepatocytes were cultured in Waymouth’s medium supplemented with 10% Fetal Bovine Serum (FBS) and 1% insulin, transferrin, selenium solution. After isolation, cells were seeded in collagen-coated plates (Biocoat, Becton Dickinson, and Bedford, MA) and rested for 4 hours and the culture medium was replaced before stimulation experiments. Primary hepatocytes were treated with thiamine at 0.1 ug/ml for 2 hours, followed by 80 mM ethanol treatment for 22 hours, in total for 24 hours. The culture medium was then collected for UA assay.

### Cell culture supernatant uric acid

The levels of UA in the culture medium of primary hepatocyte were determined using a commercial UA assay kit (Abcam, Cambridge, UK) according to the manufacturer’s protocol. UA level was measured using a colorimetric (at **λ**=570 nm) method.

### Statistical analysis

One-way ANOVA was used to evaluate demographic and drinking history measures. Univariate analysis of covariance (ANCOVA) was used to evaluate differences in the serum uric acid levels in both the groups and by the modifiers of ALD, primarily by sex (as factors) within each of the two liver injury groups. Drinking history and other demographic factors were tested as confounders (covariates) of the extent and progression of liver injury. Linear regression analysis was used to characterize the association of liver injury markers and SUA independently (or with covariables in the context of drinking history measures, sex, cytokines, and gut permeability factors as multivariable analyses). Repeated analyses of variance were performed to evaluate treatment effects of the detox program and thiamine intervention on lowering SUA. To eliminate possibility of type I error, Receiver Operating Characteristic (ROC) analysis and area under the ROC (AUROC) were used to estimate the probability of outcome of treatment in group 2 patients who reported SUA with clinically relevant SUA compared to those without at the end of the study. For subset analysis, the only additional statistical model used was two-way repeated ANOVA. Single-tail t-test was performed for the IL-17 and IL-22 mRNA expression analyses. SPSS 27.0 (IBM Chicago, IL) and Microsoft 365 (MS Corp, Redmond WA) were used for statistical analysis and data computation. Statistical significance was established at p ≤ 0.05. Data are expressed as M ± SD (Mean ± standard deviation), unless otherwise noted.

## RESULTS

### Demographics and drinking profile

There were no significant differences in the demographic measures (age and BMI) between the two groups in this study. The BMI category of the patients was overweight (>25 units) in both the groups. Males outnumbered females in group 2 (4.5-fold compared to females). The heavy drinking measures HDD90 (by 10.6%), and NDD90 (by 11.4%) were numerically higher in group 2 compared to the group 1. Lifetime Drinking years (LTDH) were significantly higher (roughly 61% more) in group 2 compared to group 1 patients as well. There was no clinical or statistically significant difference in the nutritional status of the patients between the two groups or by sex between or within each group (Table 1).

### Liver injury status in AUD patients

**Figure 1:**
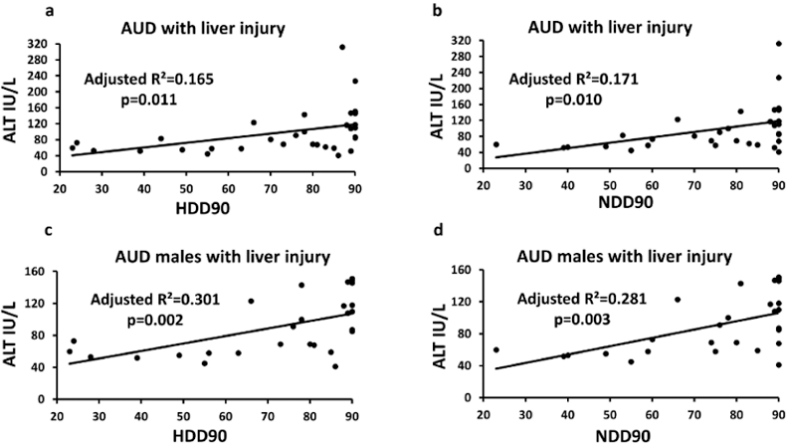
Association of heavy drinking markers with liver injury markers in alcohol use disorder patients. a) association of alanine aminotransferase and Heavy Drinking Days (HDD90) in group 2 (all AUD patients with liver injury); b) association of ALT and Number of Drinking Days (NDD90) in group 2 (all AUD patients with liver injury); c) association of ALT and HDD90 in group 2 males (all male AUD patients with liver injury); d: association of ALT and HDD90 in group 2 males (all male AUD patients with liver injury).

In group 2, ALT, AST and AST: ALT ratio values were numerically higher in females compared to the males; among these measures AST (p=0.002), and AST: ALT ratio (p=0.015) values were statistically significant. Mean AST: ALT ratio in both groups was less than 1.5, suggesting no ongoing progression of ALD; however, AST: ALT ratio values in group 2 females were more than 1.5. This suggested that the progression of liver injury was ongoing. ALT was significantly associated with NDD90 (p=0.010), and HDD90 (p=0.011) in group 2 patients. In group 2 males, timeline follow back measures and liver injury marker ALT showed significant association (HDD90: p=0.002; and NDD90: p=0.003). Liver injury marker AST also showed similar association with HDD90 (p=0.007); and NDD90 (p=0.017) (data not plotted). No such association was found in group 2 females. No other drinking measures showed any association with liver injury either in group 1 or group 2 (Figures 1a-1d).

Effect sizes are analyzed as adjusted (model-fit). Statistical significance was set as p ≤ 0.05.

### Serum uric acid characterization in AUD patients

We found a clinically significant hyperuricemia (elevated serum uric acid) level in 22 out of 48 AUD patients. Mean SUA values in group 1 AUD patients were lower than the clinical range; however, mean SUA was clinically significant in the group 2 AUD patients. Elevated SUA in both group 1 and group 2 was primarily due to reports on males. 16 out of 27 group 2 male patients (approximately 60%) exhibited elevated SUA compared to females (1 out of 6, 16.7%). Only two female AUD patients in group 1 and one female in group 2 had clinically relevant SUA levels.

### Association of serum uric acid and liver injury in AUD patients

**Figure 2:**
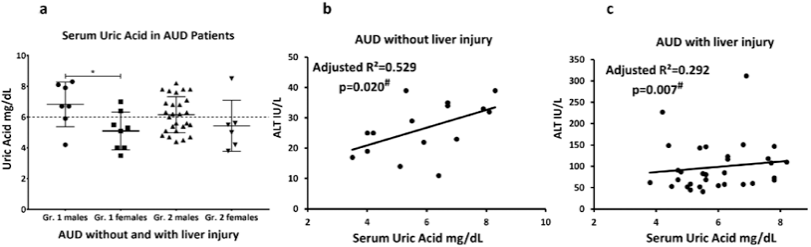
Baseline assessment of serum uric acid levels in alcohol use disorder patients, and association with liver injury (alanine aminotransferase). 2a) level of SUA in group 1 (AUD without liver injury) and group 2 (AUD with mild liver injury) distributed by sex (data presented as M ± SD); 2b) association of serum uric acid and serum ALT in group 1 (AUD patients without liver injury); 2c) association of serum uric acid and serum ALT in group 2 (AUD patients with liver injury).

ALT and SUA showed marginally significant univariate association in group 1 (adjusted R^2^=0.209, p=0.049) that augmented in effect with multivariate association with the inclusion of TNF**-α** and HDD90. Whereas in group 2 this association was not significant in univariate regression analysis (with covariable as HDD90, p=0.020). We repeated the same statistical test, only this time including covariables that are pathway specific measures of the proinflammatory activity and heavy drinking markers. TNF-**α** along with SUA and HDD90 showed significant prediction for ALT in group 2. The same test in group 1, with TNF-**α** along with SUA and HDD90, significantly predicted ALT levels, suggesting ongoing processes are still relevant even in AUD patients without liver injury. A similar response was also observed with respect to IL-1**β** in both the groups. IL-1**β** along with uric acid and HDD90 show significant albeit low effect of association with ALT (adjusted R2=0.171, p=0.049) in group 2 as well as in group 1 (adjusted R2=0.648, p=0.020, data not plotted) (Figures 2a-2c).

Covaried with HDD90 and TNF-**α**. Statistical significance was set as p ≤ 0.05.

### Association of serum uric acid and gut barrier dysfunction; and inflammation in AUD patients

SUA was significantly associated with LPS (adjusted R2=0.229; p=0.003); and LBP (adjusted R2=0.139; p=0.022) only in group 2 (data not presented figuratively). Further, we further performed a stepwise analysis of measures (as independent or covariables) that participated in gut-derived cytokine response to estimate role of proinflammatory activity. SUA showed positive significant association (low effect) with IL-1**β**, r=0.473 when covaried with LBP in group 2, there was no such association in group 1 (data presented from our group in a different publication as cited here) [24].

**Figure 3:**
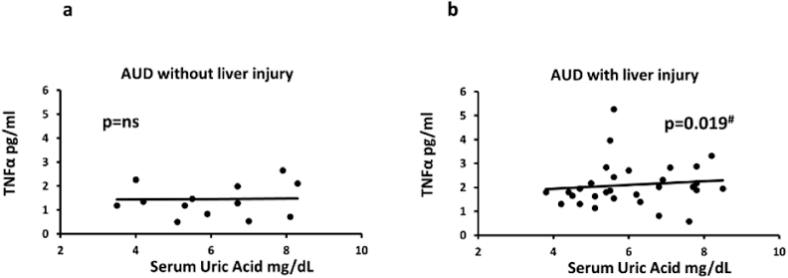
Association of serum uric acid and candidate cytokine response in alcohol use disorder patients.3a) association of SUA and TNF-α and SUA in group 1 (AUD without liver injury); 3b) association of TNF**-α** and SUA in group 2 (AUD with liver injury).

We evaluated one of the mechanisms of liver injury, namely neutrophil infiltration, by following the upstream regulation of proinflammatory activity through monocyte activated 1L1**β** response. We found that a feedback upregulation of TNF-**α** can occur through other cytokines, such as IL-6 and IL1**β**. We also identified a close association of absolute neutrophil count and SUA (covaried with IL-1**β** and absolute monocyte count), having an r=0.537 (adjusted R^2^=0.206 at p=0.029) in group 2, supporting involvement of SUA in the pathway response. This finding was also supported by a significant association (r=0.623) of SUA and TNF-**α** when covaried with IL-6, IL-1**β** and LBP (adjusted R^2^=0.282 at p=0.019) in group 2 patients, while no such association was present in group 1. Progressive increase in correlation supported the likelihood of the pro-inflammatory activity at each level of pathway response. None of these stepwise analyses yielded any significance, group 1 (Figures 3a and 3b).

Covaried with LBP, IL-1**β** and IL-6. Effect sizes are analyzed as adjusted (model-fit). Statistical significance was set as p ≤ 0.05.

### Association of cytokine and neutrophil response in liver cell death markers

IL-1β along with SUA and HDD90 show significant association with K18M65, r=0.544 in group 2. On adding absolute neutrophil count, a slightly augmented association was observed, r=0.554 (adjusted R2=0.196, p=0.050) in group 2. In the context of TNF-α and SUA, K18M65 was also significantly correlated, r=0.713. When absolute neutrophil count was included in this test, we found an even greater relationship, r=0.740 (adjusted R2=0.475, p ≤ 0.001). There was no such association found in group 1 for all above mentioned analyses (Table 2a).

K18M65:M30 (ratio) and IL-1**β** (along with SUA and HDD90) similarly showed significant association in the same statistical test, r=0.543; however not much change was observed when absolute neutrophil count was included in this test, r=0.547, p=0.056 in group 2. TNF-**α** (along with SUA and HDD90) was significantly associated with K18M65:M30, r=0.541. Again, when absolute neutrophil count was included in this test, we did not find much difference in the association (r=0.543, p=0.060). No associations were found in group 1 for all above mentioned analyses.

Association of K18M30 and IL-1**β** (along with SUA and HDD90) was not significant; and no effects were found with absolute neutrophil count. However, TNF-**α** (along with SUA and HDD90) was significantly associated with K18M30 (r=0.658, p=0.002); when absolute neutrophil was included in this test, we did not much difference in the association (r=0.688, p=0.002). Again, none of these associations were found in group 1.

### Treatment efficacy of detox and medical management with thiamine on serum uric acid

**Figure 4:**
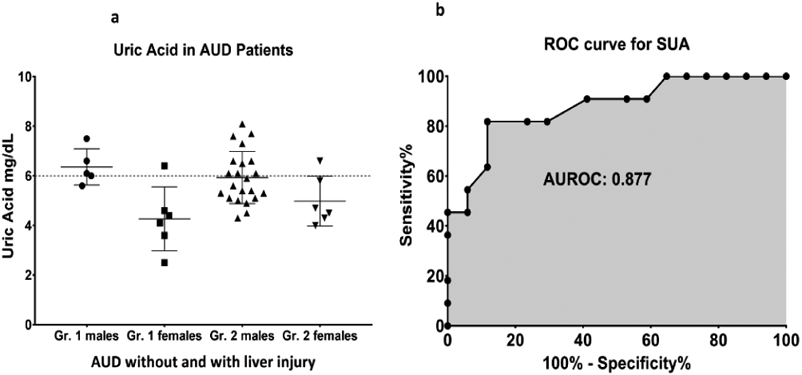
Post-treatment assessment of serum uric acid levels in alcohol use disorder patients, and association with liver injury (Alanine Aminotransferase, ALT). 4a) level of SUA in group 1 (AUD without liver injury) and group 2 (AUD with mild liver injury) distributed by sex (data presented as M ± SD); 4b) area under the “receiver operating characteristic” curve for SUA between group1 (AUD without liver injury) and group 2 (AUD with mild liver injury).

At 3-week of treatment (supervised inpatient detox [alcohol abstinence], and medical management including thiamine as treatment, SUA dropped to non-clinical levels in group 2 at post-treatment stage (5.73 ± 1.1 compared to 6.03 ± 1.3 at baseline) [25]. In group 2, 45.45% patients exhibited clinically significant SUA at baseline; this prevalence reduced to 39.3% at the end of study. Both group 1 and group 2 showed drops in SUA levels at day 22 compared to the baseline values (significant main effect of SUA, p=0.005). Males of group 2 had clinically non-significant values of mean SUA (Figures 4a and 4b).

An observed AUROC of 0.5202 supports valid discrimination of the two groups by SUA even at the early stages of ALD. Data presented as M ± SD. Statistical significance was set as p ≤ 0.05. ROC analysis performed on group 2 patients who reported SUA levels as ≤ 6 or >6 at post-treatment assessment showed that area under the ROC curve (AUROC) was 0.877 (p ≤ 0.01) with sensitivity of 81.82 and specificity of 76.47 at the baseline SUA level of 6.1 mg/dl.

### End-of-treatment changes in liver cell death/injury and serum uric acid

**Figure 5:**
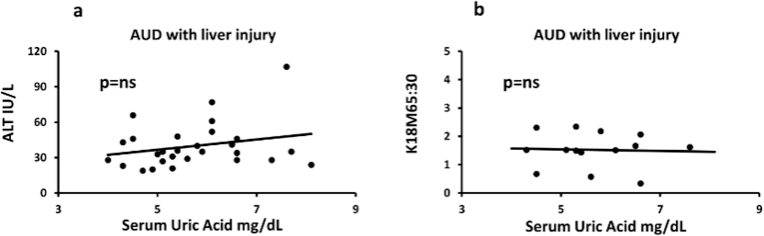
Association of serum uric acid and liver injury and liver cell death marker at post-treatment stage in group 2 patients. 5a) association of SUA and ALT in group 2 patients at week 3; 5b) association of SUA and K18M65:M30 ratio in group 2 patients at week 3.

Liver injury marker, ALT lowered to 44.71 ± 23.53 at the 3-week assessment in group 2 patients compared to their pre-study values (98.6 ± 56.4). K18M65:M30 ratio values correspondingly also dropped to non-clinical levels in comparison to their clinically significant levels at baseline in group 2. K18M65 also lowered and was comparable to the baseline values of group 1. There was also corresponding significant drop observed in K18M30 values in group 2 compared to their baseline values (Figures 5a and 5b).

Multivariate test for both sub-figures included HDD90 along with candidate cytokines independently. Association of effect sizes are analyzed as adjusted (model-fit). Statistical significance was set as p ≤ 0.05. Importantly, post-treatment levels of serum uric acid, cytokines and drinking markers on liver cell death and injury markers showed significant alleviation. Multivariate regression analyses showed that SUA along with other significant contributors of pathology did not show any statistical effects or significance following treatment. ALT and SUA did not have any significant univariate or multivariate (with HDD90 and/or cytokines) association in group 2 patients. Similarly, the significant association in SUA and M65:30 ratios that were observed in group 2 patients at baseline were not present at the post-treatment stage. This observation did not reach any significance with the involvement of each cytokine or along with HDD90, suggesting exhaustion of the role of HDD90 from abstinence at post-treatment stage. Non-clinical levels of SUA and levels of K18M65 and K18M30 showed significant association in group 2, likely indicating the normal course of the liver cell death markers at post abstinence (detox) and treatment with thiamine, which was not found at the baseline assessment. Group 1 patients did not show such post-treatment response likely due to mostly unchanged response of liver death markers. Experimental model for treatment efficacy of thiamine on IL-17 and IL-22 activity and primary hepatocyte response (Table 2b).

**Figure 6:**
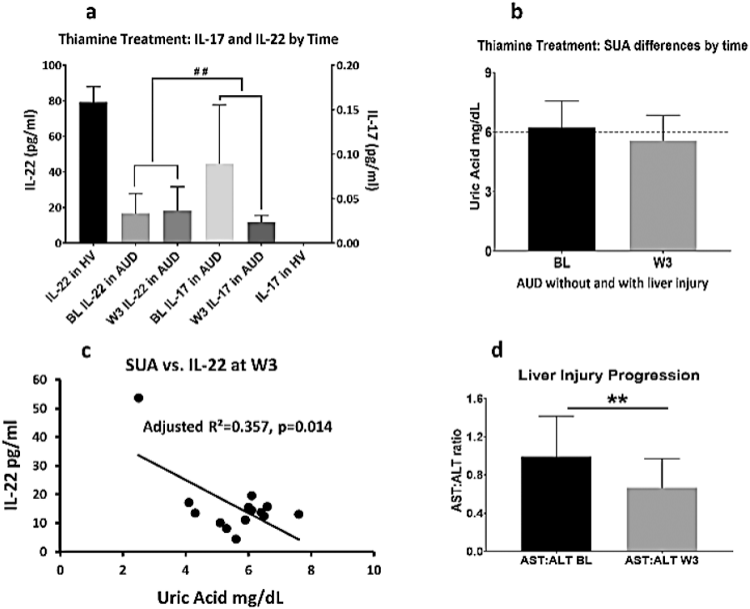
Efficacy of thiamine treatment in the production of IL-17, IL-22; and association with the changes in the serum uric acid levels with treatment. 6a) levels of IL-17 and IL-22 in healthy volunteers at baseline and in the subset analysis of AUD patients (n=16) at baseline and at week 3; 6b) changes in the SUA with thiamine treatment over the 3 weeks; 6c) significant inverse association of SUA and IL-22 at week 3; 6d) liver injury progression was significantly reduced with thiamine treatment by time.

To develop a model for thiamine activity on the proinflammatory response, we used the mechanism of IL-17 and IL-22 cytokines expression (showing proinflammatory and anti-inflammatory mediating effects respectively), which was produced simultaneously by the T-helper 17 cells [26]. In a sub-set analysis of the AUD cohort of this study (n=16), we found that the difference in the lowering of IL-17 compared to the increase in IL-22 at week 3 analysis was statistically and numerically significant. As controls, healthy volunteers showed anticipated higher expression of IL-22 and negligible expression of IL-17, showing no pro-inflammatory activity. The same sub-set cohort showed the corresponding efficacy of thiamine in lowering SUA from a clinically significant level (> mg/dL) to the normal range. To identify if the changes in SUA and cytokine responses were connected, we performed a regression analysis. The association of IL-22 (plays a protective role for liver health) was significant with SUA at medium effect at week 4 assessment; however same evaluation did not show any significance at baseline. Importantly, in the multivariate analysis, when we added IL-17 to the same statistical model, we found similar medium effect that was also significant. Lastly, in the same sub-group, AST: ALT ratio, that shows progression of liver injury, significantly dropped at the end of the treatment. When we reviewed this drop in AST: ALT ratio at treatment-end in the context of uric acid, the effects were an R^2^=0.191 that augmented to R^2^=0.443 (p=0.040) with IL-17, and to a very strong R^2^=0.614 (p=0.019) when IL-22 was added to the model (Figures 6a-6d).

Clinical levels at baseline alleviate to non-clinical (normal range). Robust significance was observed in the reverse response with treatment between IL-17 and IL-22 from baseline to post-treatment in 6b. Medium effect size of the association was observed. Data presented as M ± SD. Association of effect sizes are analyzed as adjusted (model-fit R^2^). Statistical significance was set as p ≤ 0.05.

**Figure 7:**
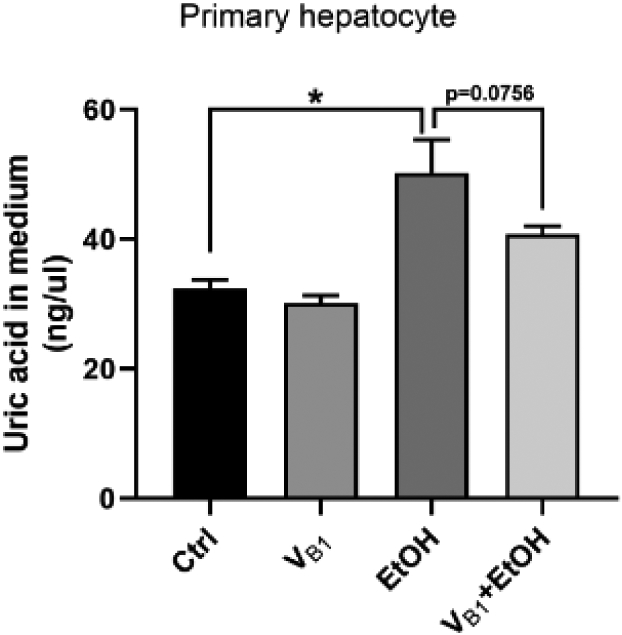
Uric acid response in the primary hepatocyte cell when exposed to alcohol (elevation) and alleviation with a low dose thiamine treatment. Ctrl: control. VB1: 0.01 ug/ml dose of thiamine. EtOH: alcohol administered. VB1+EtoH: Thiamine and alcohol administered. Data presented as M ± SD. Statistical significance was set as p ≤ 0.05.

Alcohol administration based IL-17 and IL-22 increases also dropped in the *in vitro* experimental design showing thiamine efficacy. Importantly, alcohol treatment induced a significant increase of uric acid level in the culture medium of mouse primary hepatocytes, which was moderately decreased by thiamine treatment (Figure 7).

## DISCUSSION

Serum uric acid was elevated in 22 out of the 48 (approximately 45%) patients. We found that most patients with elevated levels were males who drank heavily (regardless of liver injury). SUA was elevated in 17 out of a total of 33 AUD patients with liver injury (approximately 50%). Alcohol intake has been reported as a risk factor for alteration in uric acid levels previously [13]. In this study, we found more than 45% of chronic heavy drinkers suffered from hyperuricemia. In AUD patients with liver injury, uric acid was clinically significant, which was not observed in AUD patients without any liver injury. AUD patients with liver injury suggested an early stage of ALD with the presentation of clinical features.

We found that serum uric acid showed significant association with liver injury markers differentially in AUD patients with and without liver injury. SUA showed corresponding positive response with ALT levels in patients without any liver injury. In AUD patients with liver injury, this association was positive in the context of heavy drinking patterns (HDD90, a Heavy Drinking Marker). Involvement of heavy drinking patterns has been reported previously to be associated with liver injury and pro-inflammatory fatty acids in early stage ALD [27]. Unique markers of heavy alcohol intake (HDD90 and NDD90) were closely associated with liver injury in early-stage ALD. This finding is consistent with our previous studies [27,28]. Alterations in uric acid have been reported in non-alcoholic liver disease [29]. However, only recently has the role of uric acid in alcoholic liver disease gained importance [30]. We have recently showed that serum UA levels were higher in mice with ALD and significantly elevated when an antimicrobial peptide, cathelicidin, was absent [24]. Our findings in the current study show how the changes in alcohol- associated hyperuricemia play an important role during the early stages of ALD, along with a likely mediation of LPS (responsible for associated oxidative stress changes) and proinflammatory cytokine production [31]. Males with liver injury show distinct changes compared to the females in this study. This could suggest that at least at the early stage of ALD, such changes are more reflective in heavy drinking males.

A recent study reported that uric acid activates TLR-induced proinflammatory cytokine production [32]. High concentrations of uric acid could influence inflammatory response by increasing IL-1**β** levels (with likely downregulation of anti-inflammatory IL-1Ra receptor antagonism). We find a corresponding positive association of IL-1**β** and uric acid along with LPS and chronic drinking history in AUD patients with liver injury. This shift in cytokine surge in early stage of ALD could be one of the pathways involved in LPS-induced proinflammatory response. In the same direction, one study reported that hyperuricaemic mice have shown a higher cytokine production upon lipopolysaccharide challenge compared to their control counterparts [33]. With the 3-week treatment, we also found that the liver cell death markers improved correspondingly along with the liver injury markers and uric acid levels.

A treatment/medical management plan that included thiamine as a therapeutic agent supported alleviation of serum uric acid levels in AUD patients regardless of if they exhibited liver injury or not. However, it seems that AUD patients who had liver injury showed corresponding lowering of proinflammatory activity and necrotic type of liver cell death. Chronic alcohol intake could alter thiamine absorption; and patients with malabsorptive conditions and renal failure may also show lower absorption or hyperexcretion of thiamine [34]. Alcohol causes hyperuricemia due to an increased in the turnover of adenine nucleotides. We show that treating AUD patients with thiamine and medical management (including detox and abstinence) could alleviate serum uric acid to the normal levels.

To mechanistically validate the role of thiamine on inflammation and uric acid, we used samples of a subset of AUD patients from this investigation only. In the sub-set analysis, we showed that there is a significant interaction effect between the lowering of IL-17 and increase in IL-22. This flip corresponds well with the lowering of uric acid in the same subset, suggesting that these changes are timely and connected. In this sub-group, the progression of liver injury as characterized by AST: ALT data significantly lowered by time with treatment supporting the protective role of thiamine in liver injury. IL-22 increases were inversely associated with the lowering of uric acid at post-treatment assessment, and at that point progression of the liver injury had also ceased. Slowing of progression of liver injury also related to the lowering of IL-17 uric acid, and to IL-22 levels. Results of our primary hepatocyte cell-line model showed that uric acid levels increased with alcohol exposure compared to the control sample. This increase can be reduced at a low dose of confirming the role of thiamine and identifying the mechanism of action [35-39].

There were limitations in this study. We did not have a control group to compare the response change in SUA with cytokines or gut barrier dysfunction markers. Even though the changes in early ALD are important, such characterization of relationships and treatment efficacy are not studied well in the advanced form of ALD (for example alcoholic hepatitis or alcoholic cirrhosis). There were fewer females in the study; and those who exhibited elevated SUA were also low in number. Certainly, the results tilted mostly towards male AUD patients; more recruitment for females could further identify sex-specific changes. A 200 mg thiamine dose has been tested for a short duration and in ALD patients who did not have advanced forms like alcoholic hepatitis and alcoholic cirrhosis, thus this study does not report any potential use of thiamine in an advanced form of ALD. Study design of this investigation is longitudinal only in disease condition compared with disease controls; however, it does not include healthy volunteers as a comparator. A potential pharmacological role of ATP involved in ALD (also a stress related disease) has not been studied yet, though it has shown efficacy as an inhibitor of PARP-1 upregulation [35]. This study has low-to-mid size participation; larger studies with the various staging of ALD are needed to further characterize the role of uric acid in liver injury with respect to cytokine response and gut-barrier dysfunction [40-45].

## CONCLUSION

Our findings support the role of serum uric acid as a potential biomarker for cytokine response and gut-barrier dysfunction in alcohol-associated early-stage liver injury. We found that males are more likely to suffer from hyperuricemia with heavy drinking. Specific drinking markers, like Lifetime Drinking History (LTDH), Heavy Drinking Days (HDD90) and (NDD90), play an important role in hyperuricemia with respect to liver injury, altered cytokine response and changes in gut permeability markers.

## Figures and Tables

**Table 1:** Demographic, drinking history, liver injury measures, serum uric acid levels, nutritional status, candidate blood panel measures, cytokine, gut-dysfunction, and cell death markers of the alcohol use disorder patients tabulated by liver injury.

Measures	Group 1 (normal ALT, group 1)			Group 2 (elevated ALT, group 2)			Between group
	Males (n=7; 14.6%)	Females (n=8; 16.7%)	Total (n=15; 31.25%)	Males (n=27; 56.25%)	Females (n=6; 12.5%)	Total (33; 68.75%)	p-value
Age (years)	38.98 ± 10.28	41.74 ± 12.91	40.54 ± 11.43	44.42 ± 9.68	44.59 ± 11.45	44.45 ± 9.83	ns
BMI (kg/m2)	29.01 ± 5.04	27.95 ± 10.31	28.45 ± 8.02	25.78 ± 3.94	26.87 ± 2.73	25.99 ± 3.73	ns
**Drinking history**							
TD90	1293.8 ± 677.4	1026.6 ± 755.9	1160.2 ± 703.3	1128.6 ± 520.3	855.8 ± 449.7	1078.9 ± 512.8	ns
HDD90	73.9 ± 16.9	56.1 ± 21.4	65.0 ± 20.7	71.7 ± 22.0	77.1 ± 17.8	72.7 ± 21.2	ns
AvgDPD90	16.8 ± 6.9	16.7 ± 8.2	16.8 ± 7.3	15.1 ± 5.5	10.5 ± 4.9	14.3 ± 5.6	ns
NDD90	76.4 ± 18.6	57.3 ± 21.0	66.9 ± 21.5	74.7 ± 19.2	79.2 ± 14.9	75.5 ± 18.4	ns
NNDD90	13.4 ± 18.3	32.7 ± 21.0	23.1 ± 21.4	15.2 ± 19.3	10.7 ± 15.0	23.1 ± 21.4	ns
LTDH	12.3 ± 7.0	9.8 ± 5.1	10.9 ± 5.9	18.9 ± 9.9	11.5 ± 9.0	17.6 ± 10.1	0.025
**Liver injury markers**							
ALT (IU/L)	31.4 ± 5.9	22.1 ± 8.9	26.5 ± 8.7	90.2 ± 56.4	136.2 ± 107.4	98.6 ± 56.4	na
AST (IU/L)	36.0 ± 23.5	33.3 ± 18.2	34.5 ± 20.1	110.7 ± 82.9	220.8 ± 130.1	130.7 ± 100.5	pO.001
AST: ALT	1.1 ± 0.6	1.45 ± 0.4	1.29 ± 0.5	1.15 ± 0.6	1.9 ± 1.0	1.29 ± 0.7	ns
**Mineral analysis**							
SLA (mg/dL)	6.8 ± 1.5	5.1 ± 1.2	5.9 ± 1.6	6.2 ± 1.2	5.4 ± 1.7	6.0 ± 1.3	ns
**Nutritional status**							
CONUT	1.29 ± 1.7	0.75 ± 1.0	1.0 ± 1.4	1.0 ± 1.1	0.83 ± 0.8	0.97 ± 1.0	ns
**Blood cell measures**							
WBC (K/uL)	6.71 ± 3.5	8.21 ± 2.6	7.5 ± 3.0	6.38 ± 1.4	5.9 ± 2.3	6.0 ± 2.2	ns
AMC (K/uL)	0.55 ± 0.3	0.52 ± 0.1	0.53 ± 0.2	0.53 ± 0.3	0.46 ± 0.1	0.52 ± 0.2	ns
ANC (K/uL)	3.93 ± 2.7	4.98 ± 2.1	4.49 ± 2.4	3.49 ± 1.8	3.89 ± 1.4	3.56 ± 1.7	ns
**Candidate cytokine response**							
IL1β (pg/ml)	0.52 ± 0.4	0.71 ± 0.6	0.62 ± 0.5	0.51 ± 0.2	0.27 ± 0.2	0.47 ± 0.3	ns
IL6 (pg/ml)	3.51 ± 4.2	2.42 ± 1.8	2.96 ± 3.2	2.99 ± 1.9	7.16 ± 5.1	3.82 ± 3.2	ns
TNF-α (pg/ml)	1.56 ± 0.7	1.35 ± 0.7	1.45 ± 0.7	1.95 ± 0.7	2.75 ± 1.5	2.11 ±0.9	0.025
MCP-1 (pg/ml)	94.15 ± 28.3	112.76 ± 67.1	103.45 ± 50.4	106.83 ± 47.7	151.54 ± 109.0	115.78 ± 65.7	ns
**Candidate gut-dysfunction markers**							
LPS (EU/ml)	0.078 ± 0.06	0.079 ± 0.05	0.078 ± 0.05	0.105 ± 0.06	0.119 ± 0.07	0.108 ± 0.059	ns
LBP (ng/ml)	595.07 ± 742.96	2039.31 ± 3360.53	1317.19 ± 2455.31	1941.18 ± 2523.99	2880.36 ± 4661.28	2092.66 ± 2886.01	ns
sCD 14 (x 106 pg/ml)	7952.38 ± 1693.19	9762.08 ± 1541.51	8917.56 ± 1813.29	9190.98 ± 1811.9	10766.38 ± 1141.78	9477.42 ± 1803.29	ns
**Liver cell death markers**							
K18M65 (IU/L)	327.19 ± 528.27	291.42 ± 310.67	308.11 ± 410.12	832.79 ± 924.05	1149.33 ± 1088.31	890.29 ± 945.63	ns
K18M30 (IU/L)	236.39 ± 156.97	521.86 ± 786.36	388.64 ± 584.36	337.89 ± 327.42	559.60 ± 380.6	378.20 ± 342.46	ns
M65:M30	1.12 ± 0.86	0.95 ± 0.69	1.03 ± 0.76	2.34 ± 1.45	1.94 ± 0.91	2.27 ± 1.36	0.002

**Table 2a:** Association of cytokines, serum uric acid and heavy drinking marker (HDD90 marker) with liver cell death markers at baseline and at 22-day assessment in AUD patients exhibiting liver injury (group 2).

Measures	Univariate		SUA and cytokine in model			SUA, cytokine and HDD90 in model		
	p-value	Adjusted R^2^	95% conf. Interval [lower- upper range]	p-value	Adjusted R^2^	95% conf. Interval [lower- upper range]	p-value	Adjusted R^2^
Necrotic marker of hepatic cell death (K18M65)								
Serum uric acid (mg/dL)	0.077							
IL1β (pg/ml)	ns	−0.022	418.547-2958.198	ns	0.005	−1742.86	0.026	0.215
IL6 (pg/ml)	ns	0.045	61.020-2664.187	ns	0.07	−870.991-1828.568	0.015	0.248
TNF-α (pg/ml)	0.003	0.25	−119.748-2075.496	0.001	0.338	−2686.285-1428.697	>0.001	0.452
MCP-1 (pg/ml)	0.012	0.178	−237.287-2246.551	0.017	0.205	−1268.325-1202.737	0.001	0.424
**Necrotic index of hepatic cell death** (**M65:M30**)								
Serum uric acid mg/dL	0.072	0.072						
IL1β (pg/ml)	ns	−0.027	1.876-6.882	ns	0.035	−0.388-5.107	0.026	0.213
IL6 (pg/ml)	ns	−0.029	1.622-6.923	ns	0.036	−0.247-5.293	0.028	0.208
TNF-α (pg/ml)	ns	−0.02	1.367-6.59 8	ns	0.061	−0.364-5.124	0.027	0.212
MCP-1 (pg/ml)	ns	−0.036	1.745-7.217	ns	0.036	−0.321-5.472	0.028	0.209
**Apoptotic marker of hepatic cell death** (**K18M30**)								
Serum uric acid mg/dL	ns	−0.005						
IL1β (pg/ml)	ns	−0.029	−24.109-790.49	ns	−0.067	−339.863-603.533	ns	0.032
IL6 (pg/ml)	ns	0.057	154.109-672.273	ns	0.022	−397.596-517.922	ns	0.098
TNF-α (pg/ml)	>0.001	0.348	−204.03-476.926	0.002	0.336	−381.294-379.403	0.002	0.368
MCP-1 (pg/ml)	>0.001	0.367	−272.457-426.669	0.001	0.343	−538.272-4199.03	>0.001	0.465

The p-value and adjusted R^2^ are obtained from univariate regression model for each of the cytokines or serum uric acid and apoptotic/necrotic markers (first analyses column). The p-value and adjusted R^2^ under “serum uric acid and cytokine in model” are obtained from multivariate regression model including apoptotic/necrotic marker, SUA and each cytokine independently (second column). The p-value and adjusted R^2^ under “serum uric acid, cytokine and drinking marker in model” are obtained from multivariate regression model including apoptotic/necrotic marker, SUA, each cytokine independently and drinking marker, HDD90 (third column). Statistical significance was set at p ≤ 0.05. Effect categorization of adjusted R^2^ are: 0.2-0.4: low; 0.4-0.6: intermediate; 0.6 and more: high.

**Table 2b:** Association of cytokines, serum uric acid and heavy drinking marker (HDD90 marker) with liver cell death markers at post-treatment.

Measures	Univariate		SUA and Cytokine in model			SUA, Cytokine and HDD90 in model		
	p-value	Adjusted R^2^	95% Conf. Interval [lower- upper range]	p-value	Adjusted R^2^	95% Conf. Interval [lower- upper range]	p-value	Adjusted R^2^
Necrotic marker of hepatic cell death (K18M65), M ± SD: 498.92 ± 885.23								
Serum uric acid (mg/dL)	0.021	0.316						
IL1**β** (pg/ml)	ns	−0.035	−6226.377	0.036	0.354	−6822.796	ns	0.291
IL6 (pg/ml)	ns	−0.064	−6272.305-242.835	ns	0.258	−6953.191-331.191	ns	0.201
TNF-**α** (pg/ml)	ns	−0.047	−6202-1871.876	ns	0.276	−6938.202-1935.653	ns	0.143
MCP-1 (pg/ml)	ns	−0.069	−5739.091-728.462	ns	0.279	−6645.998-974.754	ns	0.221
**Necrotic index of hepatic cell death** (**M65:M30**), **M ± SD: 1.47 ± 0.6**								
Serum uric acid (mg/dL)	ns	−0.081						
IL1**β** (pg/ml)	ns	−0.065	−1.06-4.496	ns	−0.179	−1.221-4.848	ns	−0.287
IL6 (pg/ml)	ns	−0.068	−1.031-4.392	ns	−0.179	−1.244-4.877	ns	−0.287
TNF-**α** (pg/ml)	ns	−0.057	−2.171-4.566	ns	−0.156	−2.397-5.087	ns	−0.261
MCP-1 (pg/ml)	ns	−0.052	−1.082-4.38	ns	−0.179	−1.41-0 5.062	ns	−0.287
**Apoptotic marker of hepatic cell death** (**K18M30**), **M ± SD: 365.9 ± 544.6**								
Serum uric acid (mg/dL)	0.012	0.37						
IL1**β** (pg/ml)	ns	−0.052	−4173.396-460.325	0.025	0.395	−4432.027-397.144	ns	0.347
IL6 (pg/ml)	ns	−0.055	−3831.902-29.418	0.05	0.313	−4285.611-39.107	ns	0.289
TNF-**α** (pg/ml)	ns	−0.045	−3766.219-992.496	0.041	0.337	−4244.933-895.972	ns	0.316
MCP-1 (pg/ml)	ns	−0.066	−3549.111-275.635	0.042	0.336	−4167.685-276.344	ns	0.303

## Data Availability

The data presented in this study are available on request from the corresponding author.

## HIGHLIGHTS

- One of the causes of uric acid elevation is heavy alcohol intake, regardless of the presence of the Alcohol-associated Liver Disease (ALD).
- Clinically significant level of uric acid is observed in the early stage of alcohol-associated liver disease, mostly in males.
- Hyperuricemia in alcohol-induced liver injury can be characterized well in the context of elevated cytokine response, and exacerbated gut-permeability.
- Abstinence and treatment with thiamine could alleviate hyperuricemia in heavy drinkers with mild or no ALD.
- Hyperuricemia can serve as a clinical determinant of both alcohol use disorder and early-stage alcoholic liver disease as a transitional biomarker.

## Abbreviations:

ALD: Alcohol-associated Liver Disease
AUD: Alcohol Use Disorder
AUDIT: Alcohol Use Disorders Identification Test
HD: Heavy Drinkers
CS: Clinically Significant
LTDH: Lifetime Drinking History
LPS: Lipopolysaccharide
LBP: Lipopolysaccharide Binding Proteins
NCS: Clinically Non-significant
Serum ALT: Alanine Aminotransferase
Serum AST: Aspartate Aminotransferase
Serum K18: Cytokeratin 18
Serum K18M65: Soluble K18 (indicative of necrosis)
Serum K18M30: Caspase-cleaved Fragment of K18 (indicative of apoptosis)
SUA: Serum Uric Acid
TLFB: Timeline Follow Back
HDD90: Heavy Drinking Day Past 90 Days
TD90: Total Drinks Past 90 Days
NDD90: Number of Drinking Days Past 90 Days
AvgDPD90: Average Drinks Per Drinking Day Past 90 Days
UA: Uric Acid
